# Stem Cell-Laden Hydrogel-Based 3D Bioprinting for Bone and Cartilage Tissue Engineering

**DOI:** 10.3389/fbioe.2022.865770

**Published:** 2022-05-17

**Authors:** Zhimin Yang, Ping Yi, Zhongyue Liu, Wenchao Zhang, Lin Mei, Chengyao Feng, Chao Tu, Zhihong Li

**Affiliations:** ^1^ Department of Orthopedics, The Second Xiangya Hospital, Central South University, Changsha, China; ^2^ Hunan Key Laboratory of Tumor Models and Individualized Medicine, The Second Xiangya Hospital, Central South University, Changsha, China; ^3^ Department of Dermatology, The Second Xiangya Hospital, Central South University, Hunan Key Laboratory of Medical Epigenomics, Changsha, China

**Keywords:** stem cell, hydrogel, 3D bioprinting, bone, cartilage

## Abstract

Tremendous advances in tissue engineering and regenerative medicine have revealed the potential of fabricating biomaterials to solve the dilemma of bone and articular defects by promoting osteochondral and cartilage regeneration. Three-dimensional (3D) bioprinting is an innovative fabrication technology to precisely distribute the cell-laden bioink for the construction of artificial tissues, demonstrating great prospect in bone and joint construction areas. With well controllable printability, biocompatibility, biodegradability, and mechanical properties, hydrogels have been emerging as an attractive 3D bioprinting material, which provides a favorable biomimetic microenvironment for cell adhesion, orientation, migration, proliferation, and differentiation. Stem cell-based therapy has been known as a promising approach in regenerative medicine; however, limitations arise from the uncontrollable proliferation, migration, and differentiation of the stem cells and fortunately could be improved after stem cells were encapsulated in the hydrogel. In this review, our focus was centered on the characterization and application of stem cell-laden hydrogel-based 3D bioprinting for bone and cartilage tissue engineering. We not only highlighted the effect of various kinds of hydrogels, stem cells, inorganic particles, and growth factors on chondrogenesis and osteogenesis but also outlined the relationship between biophysical properties like biocompatibility, biodegradability, osteoinductivity, and the regeneration of bone and cartilage. This study was invented to discuss the challenge we have been encountering, the recent progress we have achieved, and the future perspective we have proposed for in this field.

## 1 Introduction

Bone and cartilage defects, a worldwide health problem leaving heavy social and family burdens, originate from diverse causes like trauma, degenerative diseases, congenital defects, tumor, and infection (osteomyelitis) (Qasim et al., 2019). More than 900 million reconstructive surgery operations are performed annually in response to all these leading causes of bone defects, far more than originally thought (Grasman et al., 2015). Bone and cartilage are two essential components of the body’s skeletal system, with the self-repair property under internal and external stimulation. But the self-remodeling process could only meet the demand of adapting to the body burden and repairing old micro-damaged tissues. Moreover, the solutions to repair bone defects including orthopedic procedures (bone grafts or substitute surgery) and medication (Gage et al., 20132018) could not implement successfully for bone and cartilage reconstruction. Therefore, the need of bone and cartilage reconstruction has assigned an extremely great market value to bone grafts and related materials (Zhang et al., 2018), while the liberalization for these orthopedic procedures must be achieved. In this case, the engineering of soft and hard tissues for the repair of bone and cartilage tissues has emerged as a trend that occupied a key factor to boost the market value (Pereira et al., 2018).

**FIGURE 1:**
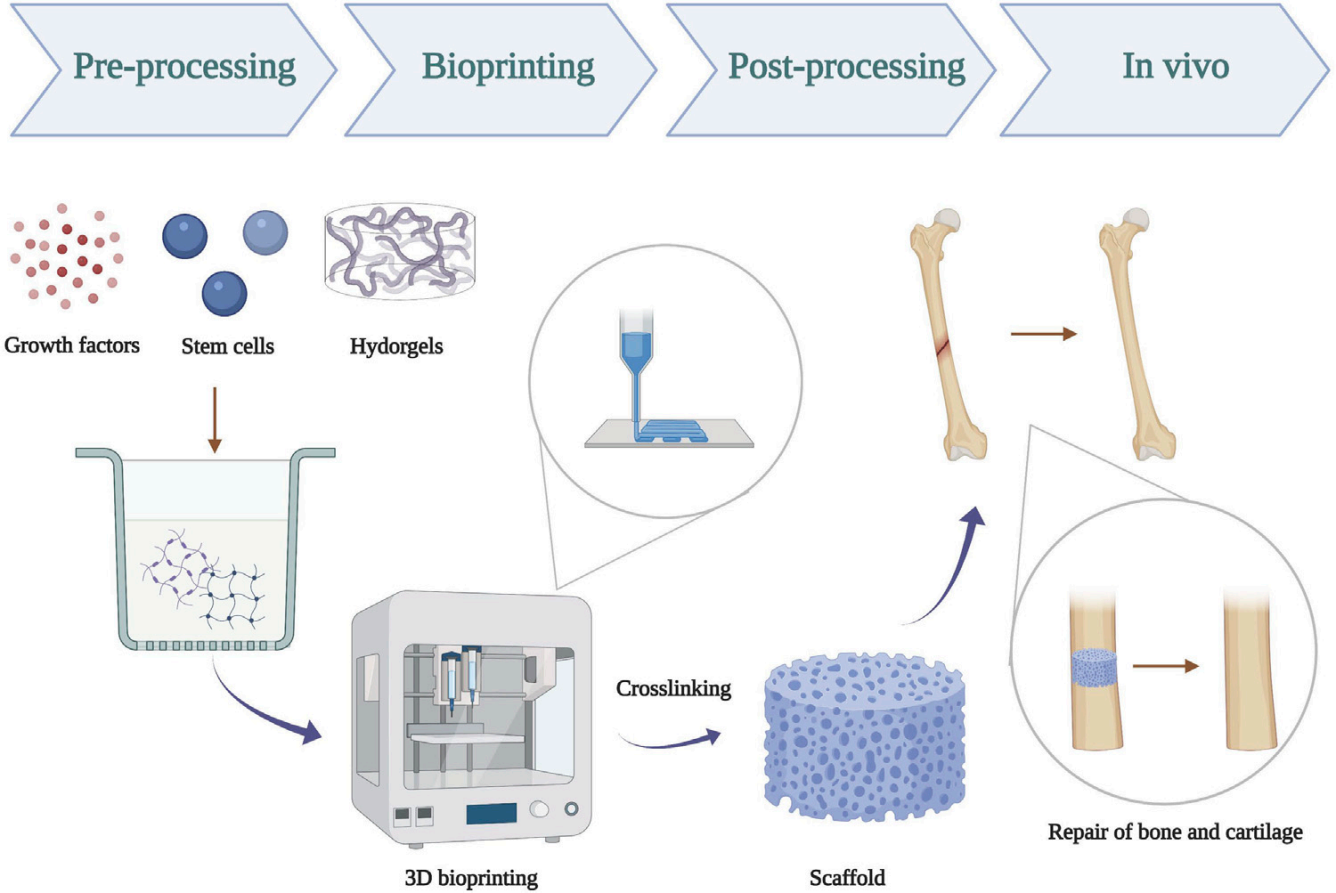
Schematic illustration of 3D bioprinting of hydrogels scaffold for repair of bone and cartilage defect. Pre-processing: prepare a mixture using hydrogel, stem cells, and growth factors; bioprinting: successful 3D bioprinting of biomaterials with physiological cell density in a designed way; post-processing: crosslinking of bioprinted constructs by UV-ray.

Three-dimensional (3D) printing was first introduced by Hull and Warfel (1986). As he described, thin layers of a material could be cured with ultraviolet light and then be printed in layers to form a solid 3D structure. Nowadays, it has been considered as a scientific hot button in the tissue engineering and biomedical field (Jain et al., 2019). Three-dimensional bioprinting is an evolutionary form of tissue engineering, which allows many cells and biomaterials to be dispensed with micrometer precision (Murphy and Atala, 2014; Moroni et al., 2018). Various 3D bioprinting technologies have been reported for the fabrication of different kinds of biological structures such as blood vessels, liver, bone, and heart (Zhu et al., 2016). Especially, scientists have produced promising prototypes of the clinically and mechanically robust bone with a functional bone marrow (Zhang et al., 2019a). Therefore, 3D bioprinting technology for bone and cartilage tissue engineering has evolved into the most promising therapeutic strategy the reconstruction (Qasim et al., 2019; Ashammakhi et al., 2019; Daly et al., 2017; Huang et al., 2019) (Figure 1). Three-dimensional bioprinting refers to using hydrogels to create complex constructs in a rapid and customizable manner, which has the capability of controlling cell distribution, high-resolution cell deposition, scalability, and cost-effectiveness. More importantly, the elements for 3D bioprinting should be characterized with low viscosity, porous, nontoxic, biodegradable, biocompatible, and promoting cell differentiation and tissue regeneration (Polo-Corrales et al., 2014; Tao et al., 2020). Hydrogels play the most essential role in 3D bioprinting because they could not only be elaborately functionalized or modified to replicate the physicochemical properties of multiple tissues (Gaharwar et al., 2014) but also provide a 3D environment similar to that of the native extracellular matrix (ECM) and deliver biological molecules like growth factors, drugs, and cells (Hayashi et al., 2009; Askari et al., 2021). Hydrogels can be composed of natural polymers and synthetic polymers. Alginate, agarose, hyaluronic acid (HA), collagen, and fibroin are representative examples for the former group, and poly(ethylene glycol) (PEG), polymer oligo(poly(ethylene glycol) fumarate) (OPF), and polyvinyl alcohol (PVA) are some salient materials for the latter group (Park et al., 2004; Mauck et al., 2006; Yamaoka et al., 2006; Markstedt et al., 2015; Hong et al., 2020; Wu et al., 2020; Zhao et al., 2020; Lamparelli et al., 2021; Liu et al., 2021). An ideal hydrogel network for bone and cartilage engineering should support cell growth/proliferation, maintain phenotypes of chondrocytes/osteoblasts, and promote chondrogenic/osteogenic differentiation of stem cells for recapitulation of the osteochondral interface or cartilage tissues (Yang et al., 2017). Bioprinted cells are crucial for correct functioning of the fabricated construct. More importantly, the cell type and the number are key important factors in bioprinting. Stem cells are a promising cell type because of their ability to proliferate in an undifferentiated but a multipotent state (self-renewal) and their capability to generate multiple functional tissue-specific cell phenotypes (Rastogi and Kandasubramanian, 2019). Modified hydrogels could mimic the microenvironment to guide the differentiation and maturation of stem cells into functional tissue constructs, which have great potential to challenge the regeneration of bone and cartilage defects by promoting osteogenesis and chondrogenesis (Xu et al., 2019a). The most commonly studied stem cells include mesenchymal stem cells (MSCs), induced pluripotent stem cells, embryonic stem cells (ESCs), and peripheral blood mononuclear cells (PBMCs) (Xu et al., 2019a; Antich et al., 2020; Guo et al., 2020; Fryhofer et al., 2021). Levato R *et al.* obtained MSC-laden polylactic acid microcarriers *via* static culture or spinner flask expansion and encapsulating MSCs in gelatin methacrylamide–gellan gum bioinks. This bioprinting approach could not only enhance the stiffness of the hydrogel constructs but also promote cell adhesion, osteogenic differentiation, and bone matrix deposition by MSCs (Levato et al., 2014). Thus, stem cell-laden hydrogel-based 3D bioprinting is a remarkable system to provide promising therapeutic strategies for bone and cartilage reconstruction.

Unlike conventional 3D printing techniques that have been used to print temporary cell-free scaffolds for use in surgery, 3D bioprinting is a relatively new technology compatible with depositing living cells. Three-dimensional bioprinting is being developed not only for transplantation but also for use in drug discovery, analysis of chemical, biological and toxicological agents, and basic research. Nowadays, this platform has been applied to bone and cartilage tissue engineering and is expected to solve the problematic issues and help to meet the future demands of cartilage and bone tissue repair. This review focused on the characterization and application of stem cell-laden hydrogel-based 3D bioprinting for bone and cartilage tissue engineering. We first introduce the current prevailing 3D bioprinting materials, techniques, and main process. Then, emphasis is put on the process and application of different subtypes of hydrogel scaffolds with various stem cells or growth factors in repairing bone and cartilage defects. Then we specifically summarize the cellular and molecular mechanisms of osteogenesis/chondrogenesis in bone and cartilage repairing. Finally, we discussed the challenges we are encountering and proposed some advice and prospects on improving the stem cell-laden hydrogel system for the field of bone and cartilage tissue bioprinting.

### 2 Strategy of Fabrication of Stem Cell-Laden Hydrogel-Based 3D Bioprinting

#### 2.1 Materials for 3D Bioprinting

The “raw materials” of bioprinting are formulations of printable biomaterials known as “bioinks” (Barrs et al., 2020). Hydrogels present immense superiority in tissue engineering and have emerged as the most common biomaterials used for 3D bioprinting. In 1954, Wichterle and Lim synthesized the first hydrogel (Ruiz et al., 2008). Nowadays, the most widely used definition for hydrogel is “hydrogel is a water-swollen and cross-linked polymeric network, produced by the simple reaction of one or more monomer/polymer/cross-linker units.” They possess a highly hydrated polymeric structure, which can be easily modified in response to various physical and biological stimuli such as temperature, light, pH, ions, and other biochemical signals (Xu et al., 2008; Gaharwar et al., 2014). As aforementioned, hydrogels can be further categorized as natural or synthetic, depending on their source. Naturally derived hydrogels originate from a biological source, with the advantage of inherent bioactivity. Generally, researchers show a preference for natural hydrogels in bone and cartilage tissue engineering because of their incomparable properties of biocompatibility, the biodegradability of hydrogel that is similar to the native bone or cartilage. Synthetic hydrogels are based on hydrated networks of polymers synthesized using chemical methods (Qin et al., 2018). Synthetic hydrogels are favored for their user-defined functionalities because they can be tailored with specific chemical and physical properties to meet the specific application requirement. Cross-linking is a key procedure in controlling these properties of the printed constructs, and various cross-linking methods have been applied for 3D bioprinting of hydrogels, such as covalent bonding, photopolymerization, thermo-gelation, cryo-gelation, and other noncovalent bonding (Huang et al., 2017). However, challenges in the use of synthetic hydrogels include poor biocompatibility, toxic degradation products, and loss of mechanical properties during degradation. Synthetic hydrogels are attractive for 3D bioprinting due to the ease of controlling their physicochemical properties during synthesis.

A wide range of hydrogels possess relevant properties and superiorities in different tissue engineering applications. Moreover, an ideal hydrogel should have proper biocompatibility, mechanical, rheological, biological, and chemical characteristics (Lee et al., 2015) because they act as a structural scaffold and provide a microenvironment for encapsulated cells (Khetan et al., 2013; Gjorevski et al., 2016). First, biocompatibility refers to the coexistence of the transplant and endogenous tissues, which is a basic design parameter for bioprinted tissues (Yilmaz et al., 2019). In addition, the hydrogel scaffold should be mechanically strong to create an environment that is compatible with cellular activities, such as cell viability, migration, and proliferation. Specially, it is important for bone and cartilage tissue engineering because these tissues mainly rely on the mechanical properties to provide solid physical support. These mechanical properties include strain, shear stress, compressive modulus, and mass swelling ratio. Moreover, scientists have made great effort to strengthen the mechanical properties of the hydrogel scaffold. For example, hydroxyapatite and graphene oxide were used to provide adequate mechanical properties for bone regeneration (Sivashankari and Prabaharan, 2020). Rheological character is the flow properties of materials under external forces, which is essential for fidelity and cell viability (Knowlton et al., 2018). Biological and chemical characteristics refer the tissue-specific modification of the printed scaffolds, which are crucial because they are related to cell growth, differentiation, and organization by direct contact. Moreover, osteoconduction for scaffold and osteoinduction for bioinks are important in bone and cartilages tissue engineering.

The choice of cells is crucial for the correct functioning of bioprinted construct. Current options for 3D bioprinting cells involve either the deposition of multiple primary cell types into patterns that represent the native tissue or printing stem cells that can proliferate and differentiate into required cell types. Stem cells are widely used because they may enhance the imitation ability of materials secondary to their unique features such as their paracrine activity and the immune-privileged status. Isolated cells can be stabilized by cross-linking during or immediately after the bioprinting process to form the final structure of the intended construct. More importantly, it is well established that the features of materials have a large influence on bioprinted cells, for example, cell attachment, size, shape, proliferation, and differentiation. Maintaining cell viability and bioactivity should be kept in sight to obtain a good biocompatibility for bioprinted constructs. In this regard, scientists have made great effort to decrease the force damages during the printing process and design an ideal scaffold to preserve cell viability. In addition, a variety of growth factors/peptides have been incorporated into bioinks to obtain superior properties for the bioprinted construct.

### 2.2 Strategies for 3D Bioprinting

**FIGURE 2:**
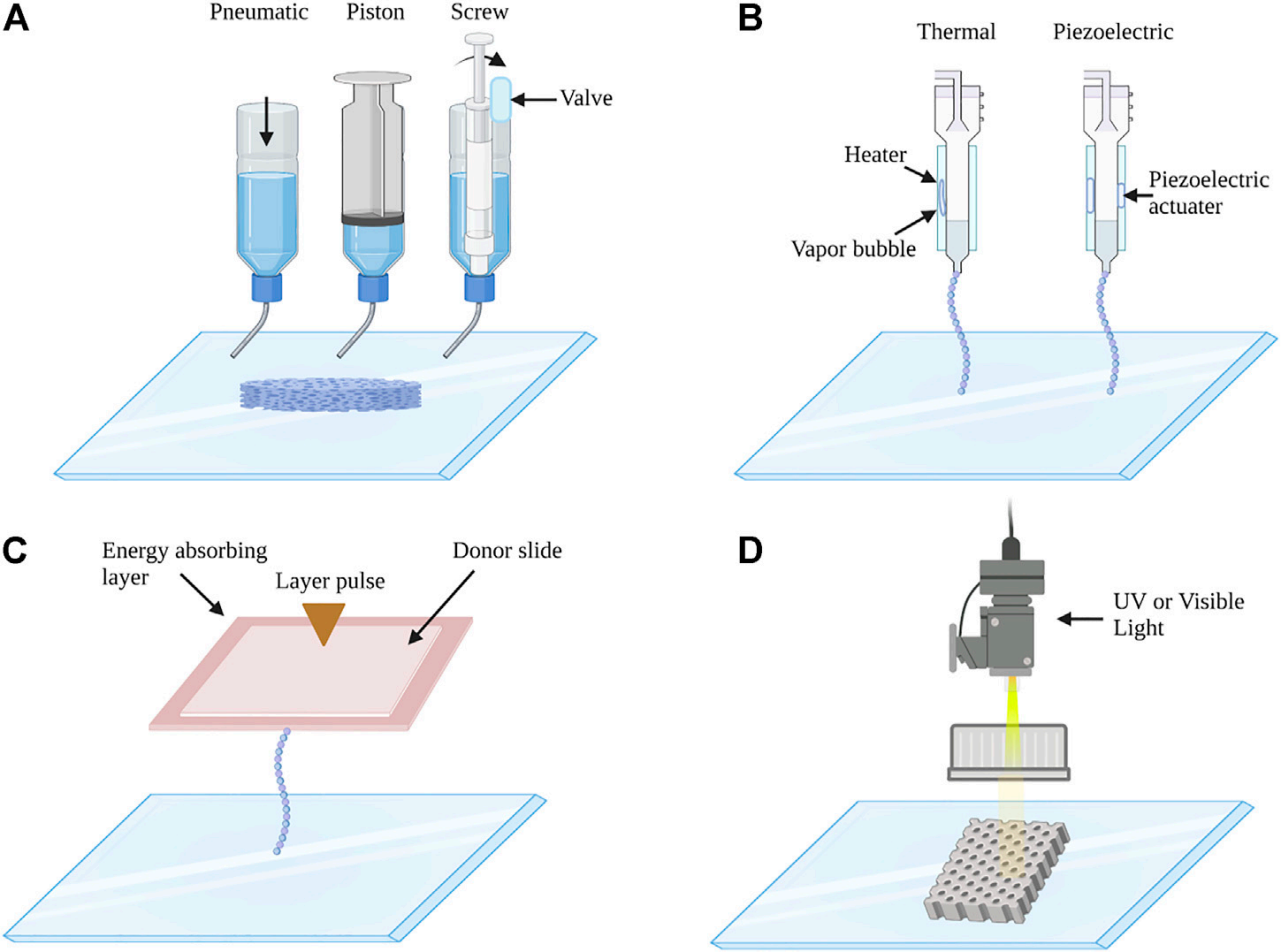
Schematic images of **(A)** extrusion-based, **(B)** inkjet-based, **(C)** laser-assisted, and **(D)** stereolithography 3D bioprinting system. **(A)** Extrusion-based 3D bioprinting: extrusion bioprinters use pneumatics, piston, or screw force to continuously extrude a liquid cell-hydrogel solution. **(B)** Inkjet-based 3D bioprinting: the printer heads are deformed by a thermal or piezoelectric actuator and squeezed to generate droplets of a controllable size. **(C)** Laser-assisted 3D bioprinting: laser bioprinters use a laser to vaporize a region in the donor layer (top) forming a bubble that propels a suspended bioink to fall onto the substrate. **(D)** Stereolithography 3D bioprinting: stereolithographic printers use a digital UV or visible light projector to selectively cross-link bioinks plane-by-plane.

Currently, 3D bioprinting techniques could be divided into four main categories: extrusion-based bioprinting, inkjet-based bioprinting, laser-assisted bioprinting, and stereolithography-based bioprinting (Figure 2). Extrusion-based bioprinting (EBB) is the most popular technique to build hydrogel scaffolds, this method uses pneumatic-, piston-, or screw-driven actuators to extrude bioinks through a nozzle onto a printing substrate. Almost all types of hydrogel pre-polymer solutions of varying viscosity as well as aggregates with high cell density can be printed with extrusion bioprinters. One of the advantages of this technique is the high structural integrity because of continuous deposition and a wide range of speed (Skardal and Atala, 2015). In addition, it has a high flexibility and enables the production of 3D bioprinted constructs with high cell density and viability (Ostrovidov et al., 2019). While beneficial, this method is limited by printing resolution, which is about 100 um (Derakhshanfar et al., 2018). Inkjet-based bioprinting consists of the thermal, piezoelectric, electrostatic, acoustic, hydrodynamic, and microvalves mechanisms, using either electrical heating to produce air-piezoelectric-pressure pulses to propel droplets from the nozzle. The drop-by-drop bioink deposition through the nozzle is synchronized with a motorized stage, allowing the fabrication of 3D constructs (Gudapati et al., 2016). The advantages of inkjet-based bioprinting include low cost, high printing speed, and high cell viability. However, it usually requires low viscosity (<10 mPa s) for bioinks (Matai et al., 2020). Laser-assisted printing is a platform that forces bioinks onto a collector substrate with pressures generated by lasers focused on an absorbing substrate (Murphy and Atala, 2014). It is a costly and fast printing technique, and it can achieve high cell viability. It allows high precision in bioink deposition; however, it has more requirements, such as rapid gelation kinetics and relatively low flow rates. Stereolithography-based bioprinting is based on light to selectively solidify bioinks in a layer-by-layer process that additively builds up objects (Moroni et al., 2018). This technology offers superior speed, resolution, scalability, and flexibility for printing a 3D structure with micrometer resolution (Ligon et al., 2017). Importantly, EBB is the most commonly used bioprinting technique in bone and cartilages tissue engineering. For example, biphasic calcium phosphate (BCP) and matrigel/alginate hydrogel composites were synthesized by EBB to induce osteogenesis of incorporated MSCs as an osteoinductive bone filler at the area of bone defects (Fedorovich et al., 2011). In addition, volumetric 3D bioprinting is a light-mediated technique, which enables excellent cell viability, structural fidelity, and tissue maturation potential (Rizzo et al., 2021). It has been reported that volumetric tomographic bioprinting of a bone model could promote osteocytic differentiation, which would be a powerful tool for biofabrication of 3D bone-like tissues (Gehlen et al., 2021).

### 2.3 3D Bioprinting Process

Bioprinters cannot print without instruction. As aforementioned, appropriate bioinks are important for creating bioprinted tissues successfully. In addition, a correct design and planning of printing paths, control the bioprinter and post-printing operations are necessary. The bioprinting process is complex and error-prone; therefore, many researchers have utilized computer-aided design/computer-aided manufacturing technologies to obtain anatomically correct tissues (Jung et al., 2016). Computer-aided design can accelerate the speed of the whole bioprinting process, and computer-aided manufacturing can guarantee the quality of what is printed (Mandrycky et al., 2016).

A fundamental step for the transition to clinical application is the good integration of various bioinks, which mimic the specific geometry of tissues of interest. The general bioprinting process is as follows (Qasim et al., 2019): designers draw the specific design taking into account the function that this structure should have *in vivo*, different operating temperatures, and appropriate printing times, in which to insert the cells by computer-aided design (Grasman et al., 2015); designers prepare and load appropriate bioinks (Gage et al., 20132018); and the bioprinter builds structures and follows the predesigned path by computer automation systems. Furthermore, post-printing modification is often used to increase or trim the scaffold performance. Such modifications not only improve differentiation and growth of stem cells *in vitro* but also enhance histocompatibility after transplantation. Therefore, post-printing is important to maintain the bioprinted structures viable. Moreover, some special approaches to fabricate tensegrity structures acting as an external stabilizations system without further process after printing, which can pave the way toward more algorithmic designs of 3D bioprinting (O’Bryan et al., 2017; Lee et al., 2020a; Chiesa et al., 2020).

Three-dimensional bioprinting could combine functions and properties of various hydrogels to generate high-resolution, multi-component living constructs, which developed exciting perspectives in the area of stem cell therapy for tissue engineering (Negro et al., 2018). Three-dimensional bioprinting represents a formidable technology in tissue engineering (Matai et al., 2020). However, a lot of problems and challenges remain to be solved. For instance, the limited materials and printing systems could not meet the stringent demand of hydrogel composite systems. We need to do further research to find an ideal material with high cross-linking efficiency. Moreover, substandard technology would bring out agglomeration of reinforcements inside the hydrogel matrix, resulting in poor performance of the hydrogel composite. Moreover, it becomes more complex to find out the most optimal bioink design in case of 3D bioprinting of bone, as it is a tough task to strike the correct balance between biocompatibility and mechanical strength. Thus, we need to take a greater effort to improve 3D printing techniques of hydrogel composites in the aspects of the material design and printing systems.

### 3 Hydrogel for 3D Bioprinting

As aforementioned, 3D bioprinting of hydrogel has been increasingly applied in tissue engineering and regenerative medicine over the past years (Yue et al., 2015; Naahidi et al., 2017). Moreover, it is a very attractive carrier for encapsulating cells because of the hydrophilic nature with the high water content, heightening the application of stem cell-laden hydrogel in the tissue engineering area (Li and Mooney, 2016). Accordingly, the ideal stem cell-laden hydrogel that is used for 3D bioprinting should possess the following capacity: 1) well printability—the ability to produce the 3D structure scaffold with high shape integrity and fidelity; 2) proper degradability—the scaffold printed and implemented in the bone or cartilage should be degraded in a speed similar to the native extracellular environment; 3) sufficient mechanical property—enough mechanical property can not only support the structure but also enhance cell viability and stimulate the differentiation of stem cells; 4) bioactivation—both *in vivo* and *in vitro* bioprinting materials should be nontoxic and no immunogenic effect to boost the cytocompatibility; and 5) differentiation of the encapsulated stem cells—the stem cells that are loaded in hydrogel should undergo osteogenic or chondrogenic differentiation with the stimuli of the mimetic environment, such as the growth factor (Schuurman et al., 2013; Li et al., 2017a; Wang et al., 2018).

However, another two intrinsic features restrict the usage of hydrogel, one is the weak mechanical property, which hampers the shape fidelity of hydrogel and the 3D printing process, the other is the performance of being prone to degradation as a bioink (Jang et al., 2018). To address these issues, the strategies have emerged to reinforce the mechanical property and bioactive feature of the biomaterial. This part overviews the recent progress of the 3D printing scaffold with stem cell-laden as the representative of the polysaccharide-based natural hydrogels (alginate, agarose, and HA) and protein-based natural hydrogels (gelatin, collagen, and silk) (Table 1).

**TABLE 1:** Hydrogel, stem cell, growth factor, cross-linking method, and 3D bioprinting method used for CTE and BTE.

Domain	Hydrogel type	Application	Stem cell type	Growth factor	Cross-linking method	3D bioprinting method	*In vivo* model	Reference
Polysaccharide	Alginate	CTE and BTE	hBM-MSCs and hAD-MSCs	-	Dual cross-linking (Ca^2+^, UV)	Extrusion	-	Olate-Moya et al. (2020), Lee et al. (2020b), Jeon et al. (2019)
			BM-MSCs, and AD-MSCs	-	CC (Ca^2+^)		Caprine and mouse	Apelgren et al., 2017, Critchley et al. (2020)
			hBM-MSCs and hMSCs	-	CC (Ca^2+^)		-	Choe et al. (2019), Ojansivu et al. (2019), Xu et al. (2019b)
	Agarose	BTE	hBM-MSCs	-	NA	Inkjet	-	Duarte Campos et al. (2016)
		CTE	hBM-MSCs	TGF-β3	Physical (temperature)	Extrusion	-	Daly et al. (2016)
	HA	CTE	hAD-MSCs	-	Double cross-linking (noncovalent bonding, Ca^2+^)	Extrusion	-	Nedunchezian et al. (2021)
			hBM-MSCs and eBM-MSCs	-	Dual cross-linking (Ca^2+^, UV)		-	Stichler et al. (2017)
		BTE	hMSCs	-	Physical (temperature)		-	Lee et al. (2018)
			hBM-MStCs	BMP-2	Photo-cross-linking (UV)		-	Poldervaart et al. (2017)
		CTE	hTMSCs	TGF-β and BMP-2	Physical cross-linking (self-assembly)		Rabbit	Shim et al. (2016)
		BTE						
Protein	Collagen	BTE	DP-MSCs	BMP-2	CC (methacrylic anhydride)	Extrusion	Rat	Fahimipour et al. (2019)
			hAD-MSCs	-	Genipin		-	Kim and Kim (2019)
	Gelatin	BTE	rBM-MSCs	-	CC (Ca^2+^), dual cross-linking (DHT, ribose)	Extrusion	Rat	Liu et al. (2020), Helgeland et al. (2021)
		CTE	hBM-MSCs	-	Physical cross-linking (self-assembly)	NA	Rabbit	Shi et al. (2017)
		CTE	hBM-MSCs	TGF-β1	NA	Inkjet	Rabbit	Han et al. (2021)
		CTE	hUCB-MSCs	-	Enzymatic cross-linking (transglutaminase)	Extrusion	Pig	Huang et al. (2021)
		CTE, BTE	raBM-MSCs	-	NA	DMD technique	Rabbit	Jiang et al. (2021)
		BTE	BM-MSCs	-	Photo-cross-linking (UV)	Extrusion	-	Dong et al. (2021)
	Silk fibroin	CTE	hAD-MSCs	-	Enzymatic cross-linking and covalent cross-linking	Extrusion	Rabbit	Li et al. (2021)
			BM-MSCs	-	Double-cross-linking (physical and chemical)		-	Ni et al. (2020)
			hMSCs	TGF-β3	CC (Ca^2+^)		-	Trucco et al. (2021)

AD-MSCs: adipose-derived mesenchymal stem cells, BMP-7D: BMP-7-derived peptides, BM-MSCs: bone marrow-derived mesenchymal stem cells, BTE: bone tissue engineering, CC: chemical cross-linking, CTE: cartilage tissue engineering, DHT: dehydrothermal, DMD: digital micro-mirror device, DP-MSCs: dental pulp mesenchymal stem cells, eBM-MSCs: equine bone marrow-derived mesenchymal stem cells, HA: hyaluronic acid, hAD-MSCs: human adipose-derived mesenchymal stem cells, hBM-MSCs: human bone marrow-derived mesenchymal stem cells, hBM-MStCs: human bone marrow-derived mesenchymal stromal cells, hMSCs: human mesenchymal stromal cells, hUCB-MSCs: human umbilical cord blood-derived mesenchymal stem cells, hTMSCs: human turbinate-derived mesenchymal stromal cells, NA: not available, rBM-MSCs: rat bone marrow-derived mesenchymal stem cells, raBM-MSCs: rabbit bone marrow-derived mesenchymal stem cells, TGF-β: transforming growth factor-beta, and UV: ultraviolet.

### 3.1 Alginate-Based Hydrogel and Their Derivatives

Alginate, a kind of polysaccharide, consists of two different uronic acids that occur naturally in the cell wall of algae and capsule of *Azotobacter* and *Pseudomonas*, and therefore can be obtained from brown seaweed and the bacterial ones (Trica et al., 2019; Ahmad Raus et al., 2020). Owing to its promising physiological properties such as biocompatibility, biodegradability, and the capability of forming gel, alginate has been widely applied in various biomedical areas, like wound dressing, drug delivery, and tissue engineering (Reakasame and Boccaccini, 2018; Rastogi and Kandasubramanian, 2019) Supplement with divalent cations such as Ca^2+^ turns the alginate into an ideal bioink for 3D printing (Song et al., 2011).

Stem cell-laden alginate-based 3D bioprinting structures have been used in different tissues including bones, cartilage, cardiac, and blood vessels (Choe et al., 2019; Chu et al., 2021; De Santis et al., 2021). However, two main disadvantages challenge the development: one is the loss of the shape fidelity and integrity after being printed due to the weak mechanical property and the other is the impairment to the adhesion and proliferation of the stem cell. To address the issues, distinct biomaterials were blended with alginate forming alginate composite to improve the property and meet the requirement of tissue engineering (Venkatesan et al., 2015; Apelgren et al., 2017; Hernández-González et al., 2020).

Compared to the pure alginate hydrogel, mixing type I collagen (COL I) and agarose into alginate could enhance the mechanical strength, while the COL I could also facilitate cell adhesion, proliferation, and expression of cartilage-specific genes (Yang et al., 2018). To improve printability and structural stability, Goeun Choe *et al.* added the graphene oxide into the alginate as a composite for bioink, revealing that the addition of graphene oxide could enhance printing and structure, viability and proliferation of MSCs, and osteogenic differentiation. As a result, the best balance of alginate and graphene oxide were identified as 3% and 0.5 mg ml^−1^, respectively (Choe et al., 2019) (Table 2). Similarly, adding graphene oxide into a tailor-made alginate-based hydrogel not only highlighted the shape fidelity and resolution of 3D scaffolds but also induced chondrogenic differentiation of human adipose-derived mesenchymal stem cells (hAD-MSCs) (Olate-Moya et al., 2020). Another attempt was to combine alginate and the decellularized extracellular matrix (dECM) derived from bone tissue of porcine, with an appropriate concentration of dECM; consequently, the printability and viability of hAD-MSCs encapsulated in the scaffold as well as the osteogenic differentiation was greatly improved (Lee et al., 2020b).

**TABLE 2:** Characteristics of various hydrogels and applications in CTE and BTE.

Hydrogel type	Origin	Constitute	Property	Disadvantage	Reinforcement material/factor	Reinforced effect	*In vivo* model	Application	Reference
Alginate	Cell wall of algae and capsule of *Azotobacter* and *Pseudomonas*	Guluronic acid and mannuronic acid	Biocompatibility	Fragility and instability	Nanofibrillated cellulose	Excellent shear thinning properties	Mouse	CTE	Apelgren et al. (2017)
			Biodegradability and low cost	Low mechanical strength, printability, and stability	Graphene oxide	Increased printability and structural stability	-	BTE	Choe et al. (2019)
			Low toxicity cross-linking ability	Low processability	Graphene oxide, gelatin, and chondroitin sulfate	Enhanced printability and anisotropic structures, cytocompatibility, and chondroinductive effect	-	CTE	Olate-Moya et al. (2020)
				Low bioactivity	Supplementing with Ma-dECM	Improved printability, cell viability, and OD	-	BTE	Lee et al. (2020b)
				Low mechanical property	OMA	Long-term storage	-	CTE, BTE	Jeon et al. (2019)
				Low mechanical property and CD	PCL	Mechanically reinforced and CD	Caprine mouse	CTE, BTE	Critchley et al. (2020)
				Weak printability	Wood-based CNF and BaG	Improved gelation and printability	-	BTE	Ojansivu et al. (2019)
				Low mechanically stability and biological supportive ability	Gelatin and PCL	Improved mechanically stability, viability, hBM-MSC proliferation, and CD	-	CTE	Xu et al. (2019b)
Agarose	Red algae	1,3-linked β-d-galactose and 1,4-linked 3,6-anhydro-α-l-galactose	Biocompatibility, high stability, low cost	Low cell adhesion	Collagen type I	Promoted cell spreading and OD	-	BTE	Duarte Campos et al. (2016)
				Poor mechanical property	PCL	Increased mechanical property	-	CTE	Daly et al. (2016)
HA	ECM of many tissues	α-1,4-D glucuronic acid and N-acetyl-d-glucosamine	Biocompatible	Poor viscoelasticity and gelation ability	Modified with biotin and streptavidin	Improved printability, shape integrity, cell viability, and chondrogenic formation	-	CTE	Nedunchezian et al. (2021)
			Antioxidant	Low mechanical stiffness and shape fidelity	MeHA	Increased mechanical stiffness, long-term stability, and OD	-	BTE	Poldervaart et al. (2017)
			Anti-inflammation	Low mechanical property and printability	Thiol-functionalized HA, P(AGE-co-G)	Increased printability, shape fidelity, and CD	-	CTE	Stichler et al. (2017)
			Chemical cross-linking	Low mechanical property and printability	Modification of CB [6] and DAH, atelocollagen, and PCL	Improved printability, CD, OD, and cartilage regeneration	Rabbit	CTE, BTE	Shim et al. (2016)
			Promote CD and proliferation	Integrity of the fabricated structures	Modified with tyramine	Increased mechanical integrity and OD	-	BTE	Lee et al. (2018)
Collagen	ECM of most tissues	Chains of polypeptide	Biocompatibility and biodegradability ECM component	Low mechanical and contraction properties	Bioceramic, modified with heparin	Increased mechanical property, elasticity, and OD	Rat	BTE	Fahimipour et al. (2019)
			Promote CD	Low mechanical property and osteogenic activity	Bioceramic (β-TCP)	Reinforced mechanical property and OD	-	BTE	Kim and Kim (2019)
Gelatin	Fishes and skins of animals and hydrolysis product of collagen	Glycosaminoglycans	Biocompatibility, biodegradability, and low immunogenicity	Weak structures and degrade rapidly	Silk fibroin	Improved mechanical properties, degradation, BM-MSC proliferation, differentiation, and ECM production	Rabbit	CTE	Shi et al. (2017)
				Low mechanical strength	HAP	Improved gelation kinetics, rheological property, and printability	Pig	CTE	Huang et al. (2021)
				Printability	PLGA	Increased printability and formability	Rabbit	CTE	Han et al. (2021)
				Low mechanical strength and poor osteoinductive ability	Nanosilicate and alginate	Improved printability, mechanical strength, and OD	Rat	BTE	Liu et al. (2020)
				Cytotoxicity of chemical of physical cross-linking	DHT and ribose	Nontoxic and CD	Rat	BTE	Helgeland et al. (2021)
				Limited cell infiltration	Methacrylate, platelet-rich plasma	Enhanced proliferation, migration, and OD and CD, M2 polarization	Rabbit	CTE, BTE	Jiang et al. (2021)
				Shape fidelity	Methacrylate and laponite nanocomposite	Improved rheological properties, the degradation stability, and the mechanical strength, BM-MSC proliferation and OD	-	BTE	Dong et al. (2021)
Silk fibroin	Silkworms and spiders	A light chain and a heavy chain linked by a disulfide bond	Biocompatibility, biodegradability, and abundant source	Limited cell growth and tissue formation ability	Tyramine-substituted gelatin	Reinforced structural stability, mechanical properties, degradation rate, stem cell aggregates, and CD	Rabbit	CTE	Li et al. (2021)
				Low mechanical property	HPMC-MA	Excellent biocompatibility and mechanical properties	-	CTE	Ni et al. (2020)
				Printability and stability	Gelatin	Printability and the elastic modulus	-	CTE	Trucco et al. (2021)

BaG: bioactive glass, BM-MSCs: bone marrow-derived mesenchymal stem cells, β-TCP: beta-tricalcium phosphate powder, BTE: bone tissue engineering, CB[6]: cucurbit[6]uril, CD: chondrogenic differentiation, CNF: cellulose nanofibrils, CTE: cartilage tissue engineering, DAH: 1,6-diaminohexane, ECM: extracellular matrix, HA: hyaluronic acid, HAP: hydroxyapatite, hBM-MSCs: human bone marrow-derived mesenchymal stem cells, HPMC-MA: hydroxy propyl methyl cellulose of methacrylation, Ma-dECM: methacrylated decellularized extracellular matrix, MeHA: methacrylated hyaluronic acid, OD: osteogenic differentiation, OMA: oxidized and methacrylated alginate, P(AGE-co-G): poly(allyl glycidyl ether-co-glycidyl), PCL: polycaprolactone, and PLGA: poly(lactic-co-glycolic acid).

In addition, supplementation with other material, especially modified alginate might be another selection. The stem cell-laden dual cross-linkable alginate microgels consist of oxidized and methacrylated alginate (OMA) directly assembled into the 3D structure and cryopreserved for long-term storage, while the stem cells could maintain equivalent after recovery compared with the freshly processed stem cells, which has provided a new paradigm for 3D printing (Jeon et al., 2019).

Although the physiology of a weak mechanical property and inferior capability for cell attachment and proliferation has affected the tissue engineering application (Ahmad Raus et al., 2020), several strategies have been explored to improve the embarrassing situation, including 1) supplementing with other materials such as graphene oxide (Li et al., 2020a) and polycaprolactone (PCL) (Critchley et al., 2020); 2) modifying the alginate-based bioink (Ojansivu et al., 2019); and 3) optimizing the fabrication method of stem cell-laden 3D-printed hydrogel (Xu et al., 2019b).

### 3.2 Agarose-Based Hydrogel and Their Derivatives

Agarose, a sort of natural polysaccharide, is extracted from red algae and composed of alternating β-d-galactopyranosyl and 3,6-anhydro-α-l-galactopyranosyl units (Krömmelbein et al., 2021). Agarose, along with its blend-based hydrogel, has been extensively used in cartilage formation and bone regeneration because of its good biocompatibility and biodegradability (Tabata et al., 2003; Zarrintaj et al., 2018). Moreover, the porous structure, tunable mechanical strength as well as the stiffness of agarose-based hydrogel facilitate the tuning of 3D scaffolds for cell culture, allowing agarose to be a promising cell-laden 3D printing hydrogel (Ulrich et al., 2010; López-Marcial et al., 2018; Salati et al., 2020).

The agarose and alginate hydrogel composites demonstrated similar effects in mechanical and rheological properties compared to pluronic hydrogel, a kind of hydrogel with well-printed capability, but exhibited better cell viability and matrix production during a 28-day culture period (López-Marcial et al., 2018). To address the problem of the dissatisfactory bioink and the limited size of the printed construct, Daniela *et al.* encapsulated the hMSC and MG-63 cells into agarose hydrogel and submerged in high-density fluorocarbon when printing, manufacturing the stem cell-laden scaffold with variable shapes and sizes, and maintaining viable cells after 21 days culture (Duarte Campos et al., 2013). To balance the contradiction between 3D printability and optimal cytocompatibility, Marius *et al.* developed a novel blend hydrogel of agarose and Col I with the encapsulation of human umbilical artery smooth muscle cells (HUASMCs), finding the blend hydrogel with a concentration of 0.5% agarose, and 0.2% Col I exhibited better stiffness, printing accuracy, and cell spreading and attachment (Köpf et al., 2016).

By contrast with native agarose, the carboxylated agarose-derived hydrogel has been regarded as a more appropriate stem cell-laden scaffold because of the significant higher human MSC survival rate (95:62%) (Forget et al., 2017). Neha Arya *et al.* explored that a human articular chondrocyte-laden extrudable carboxylated agarose-derived hydrogel, and discovered that the stiffness of carboxylated agarose-derived hydrogel and integrin-binding peptide sequence (GGGGRGDSP) could affect chondrocyte differentiation, indicating carboxylated agarose served as high suitable bioink in the cartilage area (Arya et al., 2019). To fabricate materials like the osteochondral interface that could imitate anisotropic tissues, Merve Kuzucu *et al.* worked out an extrusion-based 3D bioprinting platform based on carboxylated agarose hydrogel, and cell centration gradient and the stiffness gradient scaffold could be printed with this bioink (Kuzucu et al., 2021), laying the foundation for the manufacture of tissue with gradients, such as cartilage.

Though agarose-based hydrogel is an ideal cell-laden 3D printing hydrogel, the immigration and differentiation of stem cells encapsulated in agarose-based hydrogel alone is difficult. To address this problem, Daniela *et al.* added Col I into the agarose hydrogel, and discovered that the addition of collagen I promoted the cell spreading and osteogenic differentiation, and the effect was more obvious with the increase of collagen I in a range (Duarte Campos et al., 2016). Beyond that, mechanical compression might be another selection to induce osteogenesis. Dynamic mechanical compression could induce osteogenic differentiation of human synovium-derived mesenchymal stem cells (SMSCs) encapsulated in agarose and maintain the cartilage phenotype, providing an approach for stem cell-laden hydrogel-based cartilage therapy was found by Ge et al. (2021). Specifically, it is easier for the MSCs seeded in 3D-printed agarose hydrogel to differentiate into hyaline-like cartilage after 28-day culturing, with the presence of the transforming growth factor-beta 3 (TGF-β3), similar to alginate, while the differentiation fate of MSCs in GelMA tended to be fibrocartilage (Daly et al., 2016), providing a reference of bioink selection when printing different cartilaginous tissues.

### 3.3 Hyaluronic Acid-Based Hydrogel and Their Derivatives

Hyaluronic acid (HA), a high molecular hydrophilic natural glycosaminoglycan, is abundant in the ECM of many tissues, as well as the most abundant component in cartilage (Fraser et al., 1997; Yang et al., 2017). HA exerts its biological functions *via* antioxidant properties, biocompatibility, the presence of cell receptors, and so on (Nedunchezian et al., 2021). HA can also facilitate cell attachment, migration, and regulate differentiation of MSCs, making HA-based hydrogel a very promising stem cell-laden material for bone tissue engineering (BTE) and cartilage tissue engineering (CTE) (Zhu et al., 2006).

However, the limited viscoelastic properties of natural HA during the bioprinting process precludes its application in the 3D-printed area, while chemical modification such as methacrylate or glycidyl methacrylate makes it suitable for cross-linking, which could meet the requirement of 3D printing. For example, the mechanical stiffness and shape stability of a modified methacrylated HA-based 3D-printed scaffold could be strengthened by photo-cross-linking, evaluated by the assessment of rheology and mechanical tests, and the cell viability of human bone marrow-derived mesenchymal stromal cells incorporated into the modified hydrogel maintained 64.4% after 21-day culturing, while the osteogenesis of human bone marrow-derived mesenchymal stromal cells could be further enhanced (Poldervaart et al., 2017). Given this, researchers applied two approaches to enlarge the usage of HA-based hydrogel and their derivatives, and were applying the cross-linking strategy to increase the mechanical property; the other was integrating biological functions into physical structures by mixture with other modifications or materials.

Swathi Nedunchezian *et al.* developed a kind of adipose-derived mesenchymal stem cell (AD-MSC)-laden HA hydrogel-based 3D bioprinting applied for chondrogenic engineering by the double cross-linked strategy (Nedunchezian et al., 2021). Specifically, AD-MSC-laden HA was partially cross-linked into HA–biotin–streptavidin (HBS) hydrogel through noncovalent bonding *via* biotin and streptavidin to enhance the viscoelastic property and shape fidelity, and then the partially cross-linked hydrogel was printed to a 3D scaffold after mixed with sodium alginate and immersed in CaCl^2^ solution subsequently to heighten the final stability through the second step ion transfer cross-linking. As a result, the AD-MSC-laden HA-based 3D scaffold exhibited improved printability, shape integrity, cell viability, and chondrogenic formation compared to the HA hydrogel. Similarly, allyl-functionalized poly(glycidol)s (P(AGE-co-G)) was added into thiol-functionalized HA as a cytocompatible cross-linker, and the chemical cross-linking could be induced by UV *via* thiol-ene coupling generated by thiol and allyl groups of the two materials. The hybrid hydrogel loaded with the 3D-printed equine or human bone marrow-derived mesenchymal stem cell (BM-MSC) scaffold demonstrated higher cell viability and promising chondrogenesis after 21 days of observation, tested by live/dead cell staining, safranin-O staining for glycosaminoglycans (GAG) and immunohistochemistry (IHC) for aggrecan, Col I, and type collagen II (Col II) (Stichler et al., 2017). However, Jin-Hyung Shim *et al.* exploited a novel 3D bioprinting multilayered mechanical stable scaffold *via* the host–guest chemistry-based strategy according to the two preprocessed hydrogel supramolecular HA and atelocollagen with human turbinate-derived mesenchymal stromal cells (hTMSCs) encapsulated, avoiding the affection to the cell viability of the chemical agent and physical stimulation during cross-linking or the post-printing cross-linking process, and the *in vivo* study of this hTMSC-laden hydrogel-based 3D-printed multilayered structures for knee articular cartilage injury of rabbits proved it to be an appropriate and promoted approach according to the gross morphology, Hematoxylin and eosin staining (H&E staining), safranin-O staining, and immunohistochemistry for Col II and type collagen X (Col X) (Shim et al., 2016).

Another disadvantage of HA-based hydrogel is the long-term gelation process. In terms of this issue, Jaeyeon Lee *et al.* produced a hybrid bioink with tyramine-conjugated for improved printability, mechanical integrity, and fast gelation while BMP-7-derived peptides (BMP-7D) and osteopontin immobilized for osteogenesis, with shorter gelation time (<200 s), higher hMSC viability encapsulated in the 3D-printed scaffold (>90%), and favorable osteogenic differentiation (Lee et al., 2018).

### 3.4 Collagen-Based Hydrogel and Their Derivatives

As the most abundant protein family of the ECM, collagen accounts for two-thirds of the dry mass of adult articular cartilage (Eyre, 2004). Specifically, Col II makes up the most proportion of the articular cartilage and is accompanied by the other minor collagens to provide the tensile strength and physical property of the matrix of the cartilage (Luo et al., 2017), while bone is made of an organic matrix that consists of about 90% of Col I, which contributes to the prominent mechanical properties (Depalle et al., 2021). Both the bone marrow-derived mesenchymal stem cell (BM-MSC)-embedded Col I hydrogel and the blend collagen (Col I/II = 3:1) hydrogel exhibited chondrogenic differentiation; however, the GAG production is higher in the blend collagen hydrogel group, suggesting the enhancement of Col II in the production of GAG *in vitro*, while histochemical staining demonstrated that the blend collagen hydrogel group manifested a favorable effect of cartilage repair of the defects in the rabbit’s femur after 13 weeks treatment (Kilmer et al., 2020). Compared to MSCs in 2D culture in a collagen hydrogel, the 3D culture exhibited a stronger capability of differentiation of MSCs into osteoblasts both *in vitro* and *in vivo* of rat (Naito et al., 2013).

Though stem cells encapsulated in collagen hydrogel are widely used in BTE and CTE, 3D printing of stem cells and collagen remains challenged, whose obstacles rely on stabilizing the soft and dynamic biomaterial, and achieving the shape fidelity of the complex scaffold required the 3D printing process (Lee et al., 2019). Researchers have developed different approaches including additive manufacturing techniques and complex structure design to improve it. To reinforce the support of the scaffold, tricalcium phosphate (TCP)-based bioceramic was added by the additive layer manufacturing technique, both the compressive strength and the compressive modulus increased, forming an ideal scaffold construct (Fahimipour et al., 2019). The water solubility and photochemical cross-linking ability of collagen derivatives were greatly improved after modified with glycidyl methacrylate, demonstrating the advantage of enhancing cell adhesion, proliferation, and promoting osteogenic differentiation of BM-MSCs, and providing another approach to fabricate stem cell-laden collagen 3D bioprinting (Zhang et al., 2019b). The mechanical stiffness and printed constructs could be improved by adding thermo-responsive agarose into Col I while the 3D-printed agarose and collagen blend hydrogels with a high collagen ratio favor the osteogenic differentiation of human bone marrow-derived mesenchymal stem cells (hBM-MSCs) in it, demonstrated by two-photon microscopy, alizarin red staining (ARS), and real-time polymerase chain reaction (RT-PCR) (Duarte Campos et al., 2016). In addition to the mechanical properties, inducing chondrogenesis and osteogenesis has been another challenge in this area. Various studies reported the impact of physical–chemical cues to hydrogel materials on differentiation (Yang et al., 2021), while the effect of 3D scaffold microstructure on stem cell differentiation remains to be seen. Yang *et al.* designed two BM-MSC-laden collagen hydrogels and named them fibrous network and porous network, respectively, according to the microstructure of collagen, and the fibrous network induced more chondrogenic differentiation of the encapsulated BM-MSCs, revealing that the microstructure of the hydrogel may be a pivotal feature for BM-MSC chondrogenic differentiation (Yang et al., 2019).

Furthermore, collagen can cover the shortage of another kind of materials according to its advantage. For example, as a promising material for bone regeneration, the application of bioactive hydroxyapatite (HAP) was limited by inefficient mineralization, mechanical instability, and incomplete osteointegration, but was greatly improved by the HAP and collagen hybrid fiber hydrogel, promoting the adhesion, proliferation, and osteogenic differentiation of rabbit BM-MSCs *in vitro* and osteoinductivity and mineralization (Li et al., 2020b).

Collagen hydrogel exhibits unique superiority based on its similarity to the ECM of the native bone and cartilage. Though some disadvantages still exist, different approaches have been developed to address the shortcoming: 1) design and process of the appropriate bioink can be mechanically modified, 2) creation and production of focus on the microstructure of the scaffold, and 3) optimized fabrication method and techniques (Kim and Kim, 2019; Marques et al., 2019).

### 3.5 Gelatin-Based Hydrogel and Their Derivatives

Gelatin is a hydrolysate of collagen, as well as a major component in most tissues including bone and cartilage (Yue et al., 2015). As a derivative of collagen, gelatin inherits its superior advantages of good biocompatibility, degradability, and bioactivity of collagen as a biomedical scaffold. In addition, the lower immunogenicity and more feasible compatibility by comparison with collagen amplify the application of gelatin-based hydrogel as a cell-laden scaffold (Lien et al., 2009; Yue et al., 2015; Gao et al., 2019). Above all, the sequence arginine–glycine–aspartic among the gelatin could interact with stem cells, hence facilitating cell proliferation, adhesion, and migration (Echave et al., 2017; Bello et al., 2020).

The instability of the physically cross-linked gelatin-based hydrogel precluded its application, while the chemical cross-linking gelatin-based hydrogel demonstrated greater potential despite the inevitable disadvantage of weakness and degradability (Sakai et al., 2009). To make up for the weak strength and rapid degradability of gelatin, silk fibroin was blended in, and the appropriate mechanical and degradable properties could be adjusted by the concentration and ratios of gelatin and silk fibroin to fit in the native cartilage environment (Rodriguez et al., 2017). By comparison, the gelatin–silk fibroin bioink with a ratio of 2:1 was proven to be favorable (Huang et al., 2014). Furthermore, to optimize the function of 3D-printed BM-MSC-laden gelatin–silk fibroin, conjugating the BM-MSC affinity peptide F7 onto the 3D-printed scaffold enhanced the BM-MSC homing *in vitro* and *in vivo*, as shown of the predominant chondrogenic differentiation of the encapsulated BM-MSCs of the scaffold (Shi et al., 2017). Here is a study that applied both of the strategies mentioned earlier. Transglutaminase cross-links gelatin to stabilize the structure to sustain the 3D scaffold, while HAP was blended to strengthen the hydrogel, then the human umbilical cord blood-derived mesenchymal stem cells (hUCB-MSC) encapsulated HAP doped, enzyme cross-linked gelatin hydrogel-based 3D-printed scaffold exhibited an effect of the scaffold enhancing proliferation and chondrogenic differentiation of hUCB-MSC *in vitro* and cartilage regeneration *in vivo*, revealing the potential application of 3D-printed gelatin hydrogels in tissue engineering (Huang et al., 2021). In addition to silk fibroin and HAP, poly(lactic-co-glycolic acid), alginate, and nanosilicate can also be addictive to improve the property of gelatin (Liu et al., 2020; Han et al., 2021).

Apart from adding other materials, modulating the modification method could be another strategy. Yu *et al.* applied melt electro-writing technology to print BMSC laden high porosity and high precision scaffolds to enhance cartilage repair (Han et al., 2021). While Helgeland et al. (2021) compared three different cross-linking methods including dehydrothermal (DHT), ribose glycation, and dual cross-linking (DHT and ribose treatments), and revealed that dual cross-linking exhibited the great advantage of promoting cell proliferation and rBMSC differentiation of 3D-printed gelatin scaffolds with promising application in cartilage tissue engineering. Mentioning modification of gelatin, GelMA, a chemical cross-linking method of grafting methacryloyl by photopolymerization, is bound to cover. The modulation can be disposed under mild conditions contributing to the fabrication of stem cell-laden GelMA hydrogel and the improved formability and downregulated immunogenicity broaden the application of GelMA (Jiang et al., 2021). A composite hydrogel composed of 15% (w/v) GelMA and 8% (w/v) laponite that demonstrated favorable printability and biocompatibility was developed, promoting BMSC proliferation and osteogenic differentiation, offering a candidate of bioink in bone tissue engineering (Dong et al., 2021).

The good biocompatibility and insufficient mechanical property of gelatin hydrogel is not absolute. A study about the effect on the physical performance of different concentration of gelatin in cell-laden 3D-printed scaffold showed that the scaffold with low-gelatin concentration (0.8% alginate) displayed good cell viability and cell morphology, same as described before, while high-concentration scaffold (2.3% alginate) preserved the scaffold shape fidelity and mechanical property but significantly impaired cell viability (Zhang et al., 2019c). In consideration of the improved physical property tunable technique, we usually focus on the low-gelatin concentration scaffold.

### 3.6 Silk Fibroin-Based Hydrogel and Their Derivatives

Silk fibroin is a fibrous protein mainly produced by silkworms and spiders, in the form of aqueous protein solution (Melke et al., 2016). Due to the remarkable mechanical property, impressive biocompatibility, and slow and tunable degradability, silk fibroin has been widely applied in tissue engineering as a biomedical material (Rockwood et al., 2011). Based on the diverse structure of the silk fibroin fabrication, the silk fibroin scaffold can be divided into films, mats, artificial fibers, sponges, and hydrogels (Yan et al., 2012; Jacobsen et al., 2017; Zhang et al., 2017; Yin and Xiong, 2018). Among them, with a 3D polymer network, hydrogel could be cross-linked physically and chemically processing for cell seeding and encapsulation, demonstrating great potential in the development of the cell-laden biomaterial area (Ahmed, 2015).

For instance, with induction of high temperature, low PH, vortexing, sonication, high ionic strength, freeze gelation, or electrogelation, silk fibroin can transform into a hydrogel form (Kim et al., 2004; Wang et al., 2008; Yucel et al., 2009; Bhardwaj et al., 2011). Though the silk fibroin hydrogel scaffold with an ideal mechanical property was processed according to the methods aforementioned, the cells encapsulated in them cannot fit in the environment of the structure produced by these techniques, as the biocompatibility was an imperative factor for processing of the cell-laden scaffold (Piluso et al., 2020). Under the circumstances, chemically cross-linking silk fibroin hydrogel maybe a better selection. For example, the degradable and biocompatible silk fibroin hydrogel have been acquired by riboflavin (Piluso et al., 2020) and HRP (Ribeiro et al., 2018). However, the physiologically fabricated silk fibroin hydrogel would lose the mechanical property and impaired the biomedical application (Jonker et al., 2012). In terms of this issue, some strategies have successfully increased the mechanical performance, such as adding functionally complementary bioink and optimizing the printing process (Kapoor and Kundu, 2016; Trucco et al., 2021).

Li et al. (2021)fabricated a silk fibroin-based 3D-printed macroporous hydrogel scaffold though HRP-medicated cross-linking of silk fibroin and gelatin under physiochemical condition, with marvelous structure fidelity, remarkable mechanical property, tunnel degradability, and a cell aggregate seeding method applied to improve the MSC inoculation efficiency in it, promoting osteogenic differentiation and articular cartilage repair in the rabbit model. Ni et al. (2020) developed a BM-MSC-laden silk fibroin hydrogel-based 3D printing with a double-network scaffold, and mechanical property including fracture strength, breaking elongation, and compressive reproducibility greatly increased with satisfied cell viability, proliferation, and chondrogenic differentiation, revealing the promise of silk fibroin for cartilage tissue engineering.

Another shortcoming of silk fibroin is the deficiency of the cell adhesion sequence resulting in poor interaction between stem cells and biomaterial (Hasturk et al., 2020). Based on this problem, combination with the collagen, poor mechanical properties and cell-mediated shrinkage could be a solution. Buitrago et al. (2018) designed a hybrid silk fibroin/collagen hydrogel, whose scaffold structure, mechanical property, and cellular behavior were greatly enhanced compared to the pure silk fibroin or collagen hydrogel.

### 4 Various Stem Cells Encapsulated in Hydrogel for 3D Bioprinting

Three-dimensional bioprinting has been a very promising approach in regeneration medicine due to the superiority of this technique with precisely control of construction of the complexed structures of tissues and organs (Murphy and Atala, 2014). However, selection of the appropriate bioink to print an ideal tissue such as bone or cartilage is still a problem that is confusing us. Hydrogel is emerging as a versatile biomaterial among the variety of biomaterials because of its good cytocompatibility, bioactivity, and degradability (Stephanopoulos et al., 2013), and various *in vitro* and *in vivo* studies have proven that the cell-laden hydrogels have opened the new possibility for reconstruction of osteochondral and cartilage tissues (Yang et al., 2017). Specifically, the cells embedded in the hydrogel can be human or animal chondrocytes, mesenchymal stem/stromal cells (MSCs), and so on, and the stem/stromal cells loaded in the hydrogel exhibited incomparable advantage due to the abundant cell sources, the multidirectional differentiated ability as well as the paracrine activities and the immune-privileged status that can imitate the heterogeneity of the tissues (Roseti et al., 2018). This part will center on the various stem cells encapsulated in hydrogel for 3D bioprinting.

### 4.1 BM-MSCs

Bone marrow-derived mesenchymal stem/stromal cells (BM-MSCs) are heterogeneous population cells with the potential of multilineage differentiation and are mainly isolated from bone marrow tissue. The BM-MSCs were first discovered and the most prevalent MSCs in clinical practice (Strioga et al., 2012). For example, Costantini et al. (2016)bioprinted a hybrid hydrogel composed of GelMA, chondroitin sulfate amino ethyl methacrylate, and hyaluronic acid methacrylate (HAMA) with high density hBM-MSCs (10^7^/ml), while the cell viability was higher than 85% demonstrated by a live/dead assay 3 h after printing, and UV cross-linking and the chondrogenic and osteogenic differentiation was ideally validated by immunocytochemistry (ICC) and RT-qPCR of aggrecan, collagen I, II, and X (Table 3). Alternatively, De Moor et al. (2020) combined spheroids of BM-MSCs with GelMA as the boink, and the viability and cartilage phenotype of the BM-MSC spheroids were maintained after bioprinting and 42 days culture, which is demonstrated by the increase in GAG, COL II, and decrease in Col I (Table 3). To enhance the osteogenesis, Wenhai Zhang *et al.* fabricated a 3D-printed BM-MSC-laden HA (Me-HA)/PCL with dual small molecule (RVS, SrRn) loaded, and both of the molecules upregulated the expression of ALP, runt-related transcription factor 2 (RUNX2), osteocalcin (OCN), and collagen 1A1 (Col 1A1) of BM-MSCs, while the osteogenic differentiation of the combination of the two molecules was more significant. Consistently, more newly formed bone can be observed in the rat bone defect model after 8 weeks implantation of the dual small molecule-loaded 3D scaffold (Zhang et al., 2020a). Challenges still remained in the clinical translation of the implantation of the stem cell-laden 3D scaffold because of the avascular structure, especially in the large osteochondral defect. To address this problem, Daly et al. (2018) developed a 3D-printed BM-MSC-laden GelMA hydrogel that guided vascularization during endochondral bone repair, revealing that this BM-MSC-laden 3D scaffold could not only facilitate vascular network formation but also promote bone formation.

**TABLE 3:** Stem cell-laden in the hydrogel and the biochemical characteristic.

Stem cell type	Species	Hydrogel	Application	Cell density/million cells*ml^−1^	Cell viability	Osteogenic/chondrogenic evaluation				
						Biochemical assay	Gene expression	Matrix synthesis	Biophysical testing	Reference
BM-MSCs	Human	GelMA, CS-AEMA, and HAMA	CTE and BTE	10	85–90%	NA	Aggrecan, Col I, Col II, and Col X	Aggrecan, Col I, Col II, and Col X (ICC)	Rheology and mechanical testing	Costantini et al. (2016)
	Human	GelMA	CTE	1	88%	GAG and Col II content	NA	GAG, Col I, Col II (IHC) H&E, PSRS, and ABS	Compression test	De Moor et al. (2020)
	Mouse	Me-HA and PCL	BTE	2	High	NA	ALP, RUNX 2, OCN, and Col 1A1	ALP staining and MTS	NA	Zhang et al. (2020a)
	Rat	GelMA	BTE	20	High	DNA, GAG, Col I II, and Col X content	Col I, Col II, and Col X	H&E, Col I, Col II, and Col X (IHC)	Compression test	Daly et al. (2018)
AD-MSCs	Human	HA and alginate	CTE	8	High	NA	SOX-9, AGG, Col I, and Col II	ARS and DMMB assay	Rheological test	Nedunchezian et al. (2021)
	Human	Alginate and Ma-dECM	BTE	5	High (>90%)	NA	ALP, BMP-2, OCN, and OPN	ARS and OPN (ICC)	Compression and rheological test	Lee et al. (2020b)
	Human	Gelatin and alginate	BTE	3	89%	NA	RUNX2, OSX, and OCN	H&E staining, MTS, OCN IHC, OCN, and RUNX2 (IF)	NA	Wang et al. (2016)
	Human	PLA nanofiber–alginate hydrogel	CTE	1.375	>90%	NA	NA	H&E, PSRS, and ABS	Compression test	Narayanan et al. (2016)
DPSCs	Human	PCL and GelMA	BTE	1	90%	NA	OPN and OCN	ARS, OPN, and OCN IF	Compression test and degradation	Buyuksungur et al. (2021)
	Human	ECM-based hydrogel and AMPs	BTE	1	90%	NA	RUNX2, COL 1A1, and OPN	ALP staining, ARS, and H&E	Mechanical test, rheological test, and printability	Dubey et al. (2020)
	Human	GelMA	BTE	NA	>90%	NA	RUNX 2, OCN, and Col 1A1	ARS and OCN (IF)	Compressive mechanical properties, swelling, and degradation	Park et al. (2020)
UVECs	Human	Alginate–gelatin	BTE	10	High	NA	OPG	VEGF and OPG (ELISA)	Compression test and stiffness test	Chen et al. (2018)
UVECs and BM-MSCs (2:1)	Human	GelMA and silicate nanoplatelets	BTE	2	NA	NA	ALP, OPN, OCN, and Col I	ARS, OCN, and Runx2 (immunostaining)	Compression test, stiffness test, and printability	Byambaa et al. (2017)

ABS: alcian blue staining, AD-MSCs: adipose-derived mesenchymal stem cells, CS-AEMA: chondroitin sulfate amino ethyl methacrylate, AGG: aggrecan, ALP: alkaline phosphatase, AMPs: amorphous magnesium phosphates, ARS staining: alizarin red staining, BM-MSCs: bone marrow-derived mesenchymal stem/stromal cells, BMP-2: bone morphogenetic protein-2, BTE: bone tissue engineering, Col I: collagen type I, COL, 1A1: collagen type I alpha 1, Col II: collagen type II, Col X: collagen type X, CTE: cartilage tissue engineering, DMMB: dimethylmethylene blue, DPSCs: dental pulp stem cells, GAG: glycosaminoglycans, GelMA: gelatin methacrylamide, HA: hyaluronic acid, HAMA: hyaluronic acid methacrylate, H&E: hematoxylin and eosin staining, ICC: immunocytochemistry, Ma-dECM: methacrylated decellularized extracellular matrix, Me-HA: methacrylated hyaluronic acid, MTS: Masson’s trichrome staining, OCN: osteocalcin, OPN: osteopontin, PLA: polylactic acid, PSRS: picrosirius red staining, RUNX 2: Runt-related transcription factor 2, sGAG: sulfated glycosaminoglycans, UVECs: umbilical vein endothelial cells, VEGF: vascular endothelial growth factor.

### 4.2 AD-MSCs

Adipose derived mesenchymal stem cells (AD-MSCs) are isolated from adipose tissue, consequently the abundant source and the large amount of the stem cells as well as the suitable biological characteristics greatly broaden the clinical application (Strioga et al., 2012; Wang and Liu, 2018). And it is known that AD-MSCs have been widely used in bioprinting organs and tissues as a bioink by virtue of the multilineage differentiation (Wang and Liu, 2018). Swathi *et al.* developed a bioink of modified HA-based hydrogel with AD-MSCs encapsulated, and printed a 3D scaffold with this bioink. MTT assay was used to assess the cell viability for 1-, 4-, and 7-day culturing, observing an increase of the AD-MSC viability laden on the scaffold compared to the non-3D-printed group. However, the expression of the chondrogenic marker gene (SOX-9, aggrecan, Col I, Col II) significantly increased at day 5 and sGAG enlarged from days 7–14 in the AD-MSCs of the scaffold, revealing the potential chondrogenic differentiation of AD-MSCs (Nedunchezian et al., 2021).

As we all know, hydrogel is usually physically or chemically cross-linked to achieve a stable structure to sustain the proliferation and differentiation of the stem cells. It must be admitted that the impair of the improper cross-linking is really lethal to the cell viability. Lee *et al.* proposed an alginate-based hydrogel encapsulated with AD-MSCs, and cross-linked via UV exposure with the dose (0–6.0 J/cm2); however, the higher dose (2.4 J/cm2) greatly suppressed the cell viability with a dose-dependent effect, while the dose of UV less than 2.4 J/cm2 exhibited high cell viability (90%) (Lee et al., 2020b). *In vivo*, the AD-MSC-laden gelatin–alginate-based 3D-printed scaffold was implanted into the dorsal area of the BALB/c nude mice subcutaneously for 8 weeks, and the results of RT-PCR, immunofluorescent staining and western blotting showed the significant increase in the osteogenic gene (OSX, RUNX 2, and OCN), demonstrating obvious bone formation (Wang et al., 2016). Though proliferation and differentiation of the AD-MSCs laden in 3D-printed hydrogel were demonstrated, they cannot last long, with a peak at day 7 and then decreased gradually during a 16-day observation (Narayanan et al., 2016).

### 4.3 Dental Pulp Stem Cells

Human dental pulp stem cells (hDPSCs) are generally isolated from the teeth that are extracted by the dentist, and demonstrate great promise in BTE because of the low-cost and easy accessibility (Fernandes et al., 2020). Buyuksungur et al. (2021) bioprinted a 3D scaffold *via* PCL and GelMA that carries hDPSCs, and then the stable composite 3D structure exhibited excellent cell viability during a 21-day dynamic test of live/dead cell assay and ideal osteogenic differentiation as well as mineralization validated by immunofluorescent staining of OPN, OCN, and alizarin red staining, respectively. To induce the osteogenic differentiation of hDPSCs *in vivo*, amorphous magnesium phosphates (AMPs) was doped in the ECM-based hydrogel, observing high cell viability, mineralization, and osteogenic markers in the absence of growth factors *in vitro* and significant increase in bone formation and bone density at bone defect of the rats skull at 4 and 8 weeks, tested by micro-CT, H&E staining, Masson’s trichrome staining (MTS), paving the way for clinical translation of the hDPSCs-laden hydrogel-based 3D scaffold (Dubey et al., 2020). Furthermore, the additive growth factor such as BMP-2 may be another strategy to induce the differentiation of hDPSCs-laden in the scaffold. Park *et al.* tethered a novel BMP peptide to GelMA-based hydrogel, and printed a scaffold with this bioink. Though nearly half of the conjugated BMP peptide missed after 3 weeks culture, the BMP peptide-tethering scaffold exhibited much more calcification than the non-BMP peptide scaffold group, suggesting a promising approach to induce differentiation of stem cells laden on the 3D structure (Park et al., 2020).

### 4.4 Umbilical Vein Endothelial Cells

Human umbilical vein endothelial cells (hUVECs) are a kind of endothelial cells isolated from human umbilical cord veins after the child’s birth (Lei et al., 2016). It is reported that HUVECs expressed many endothelial biomarkers related to the vascular homeostasis, thereby HUVECs were applied into angiogenesis as well as vascularization of tissue engineering, such as bone tissue (Kocherova et al., 2019). HUVECs are the most common endothelial cells explored as a biomaterial and have been successfully differentiated into 3D structure alone or cocultured with other cells (Bersini et al., 2016; Kocherova et al., 2019). Chen et al. (2018) fabricated a hybrid scaffold *via* HUVECs-laden alginate–gelatin hydrogel and polydopamine-modified calcium silicate (PDACS)/PCL, finding a high viability, increased proliferation as well as angiogenesis and osteogenesis of HUVECs encapsulated on the scaffold. In fact, it is the angiogenesis of hUVECs that could couple with the osteogenesis of other kind of stem cells rather than the direct osteogenic differentiation of hUVECs made it applied in bone tissue engineering. For example, to fabricate a complex bone construct with vasculature that mimic the large-scale bone tissue, Byambaa et al. (2017) cocultured hUVEC and hBM-MSCs with a ratio of 2:1 in the GelMA-based hydrogel before bioprinting, then formed a capillary-like network inside the printed construct rely on the synergistic interactions of the cocultured hUVEC and hBM-MSCs in the system, leading to a hopeful approach for the treatment of vascularized bone tissue regeneration.

## 5 Growth Factors/Peptides Promote Osteogenesis and Chondrogenesis of Stem Cells That Laden on the 3D-Printed Hydrogel

### 5.1 Mechanisms of Growth Factors on Osteogenesis and Chondrogenesis

**FIGURE 3:**
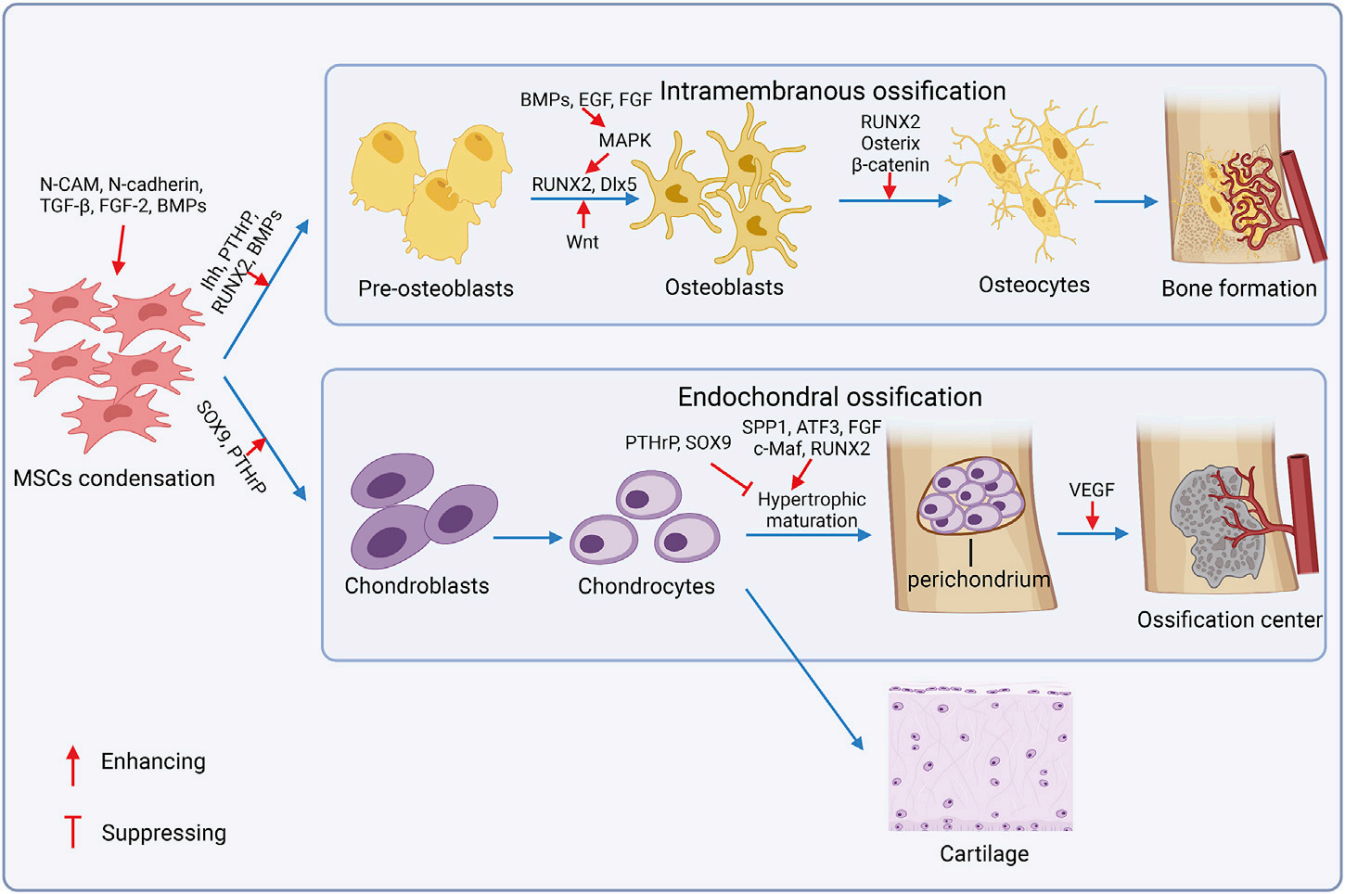
Mechanisms of osteogenesis and chondrogenesis. In bone remodeling sites, MSCs aggregate and form mesenchymal condensations. Then, bone is formed in two ways: intramembranous ossification and endochondral ossification. During the intramembranous ossification process, MSCs are differentiated into pre-osteoblasts, then they lost proliferation capacity and mature into osteoblasts, which secret alkaline phosphatase and osteocalcin that participate in the secretion, maturation, and mineralization of the extracellular matrix (ECM). In endochondral ossification, differentiated chondrocytes either proliferate in cartilage elements or exhibit hypertrophic maturation for subsequent endochondral ossification.

In bone remodeling sites, MSCs renew and proliferate, and then are committed to adipogenesis, chondrogenesis, and/or osteogenesis. Osteogenesis occurs in the intramembranous and endochondral ossification pathway (Berendsen and Olsen, 2015), and chondrogenesis precedes endochondral ossification (Figure 3). MSCs aggregate and form mesenchymal condensations through the interaction with surrounding epithelial cells and ECM components like syndecan, tenascin, fibronectin, and versican (Choocheep et al., 2010). Neural cell adhesion molecule (N-CAM) and neural cadherin (N-cadherin) are required for mesenchymal condensations and chondrogenesis (Delise and Tuan, 2002). Growth factors also stimulate condensation and overt chondrogenic differentiation, including the tumor growth factor-beta (TGF-β) subfamily (Jin et al., 2010), fibroblast growth factor 2 (FGF-2) (Zhang et al., 2020b), and bone morphogenetic proteins (BMPs) (Jin et al., 2006). Chondrocytes proliferate and form a perichondrium surrounding condensations, secret type 2a1 collagen, and the aggrecan-rich matrix to enlarge the cartilage before hypertrophy and terminal differentiation, and express specific genetic program controlled by the transcription factors SRY box transcription factor 9 (SOX9) and runt-related transcription factor 2 (RUNX2). SOX9 is required in chondrocyte differentiation, and the deficiency of SOX9 exhibits severe chondrodysplasia (Akiyama et al., 2002). RUNX2 expressed in pre-hypertrophic chondrocytes promotes chondrocyte hypertrophy; however, highly-expressed RUNX2 in perichondrial cells exerts antagonistic function on chondrocyte proliferation and hypertrophy(182). Additionally, homeodomain (HOX) transcription factors (BARX2, NKX3-2, MSX1, MSX2, PAX1, and PAX9) (Hinoi et al., 2006), TGF subfamily (Pizette and Niswander, 2000), insulin-like growth factors (IGFs) (Oh and Chun, 2003), and FGF (Danišovič et al., 2012) are also crucial for chondrogenesis. Differentiated chondrocytes either proliferate in cartilage elements or exhibit hypertrophic maturation for subsequent endochondral ossification. Articular chondrocytes secrete aggrecan and lubricin for joint smoothness and low level of collagen 2 with the expression of SOX9, SOX5, and SOX6 but the switch off of RUNX2 expression (Lefebvre and Smits, 2005). Hypertrophic chondrocytes mineralize the surrounding matrix, secrete the vascular endothelial growth factor (VEGF) and other factors to attract vessels and chondroclasts, and induce the transforming of perichondrial cells to osteoblasts, which is regulated by a series of cell and stage-specific molecular pathways, called endochondral ossification. Osteogenic molecular pathways regulate chondrocyte pre-hypertrophy, including the Indian hedgehog-the parathyroid hormone-related peptide (*Ihh–PTHrP*) axis, BMPs, Wnt-β-catenin canonical pathway, and MAPK signaling pathway (Deschaseaux et al., 2009). Mechanically, Ihh, PTHrP, and BMPs synergistically promote osteoblastic differentiation through inducing RUNX2 and downstream of RUNX2-Osterix (Krishnan et al., 2001). Conversely, PTHrP suppresses hypertrophic maturation to control bone formation in a negative feedback manner. The Wnt pathway stimulates proliferation, promotes osteoblast differentiation, and inhibits adipogenesis or chondrogenesis potential. BMPs and growth factors like the epidermal growth factor (EGF) and FGF can trigger the MAPK pathway that facilitates osteoblast maturation by phosphorylating the osteoblast-specific transcription factors and enhancing the function of RUNX2 and Dlx5 (Ge et al., 2007; Ulsamer et al., 2008). Notably, SOX9 serves as a negative regulator of chondrocyte hypertrophy (Akiyama et al., 2004), and RUNX2 emerges as a key effector of hypertrophic maturation (Dong et al., 2006). Finally, chondrocytes undergo terminal differentiation after the hypertrophy stage under the facilitation of activating the transcription factor 3 (ATF3) (James et al., 2006), RUNX2, and c-Maf (MacLean et al., 2003), and increase the expression of matrix metalloproteinase 13 (MMP13), osteopontin (spp1), and alkaline phosphate (Adams and Shapiro, 2002).

During the intramembranous ossification process, MSCs are differentiated into pre-osteoblasts, then they lost proliferation capacity and are matured into osteoblasts, in which secret alkaline phosphatase and osteocalcin participate in the secretion, maturation, and mineralization of ECM, under the mutual exclusive and fine-tuned control of transcription factors (Chen et al., 2016; Infante and Rodríguez, 2018). Eventually, osteoblasts either become osteocytes or inactive bone-lining cells (BLCs), or die by apoptosis. The transcription factors, such as RUNX2, osterix, SP7, and β-catenin, have played a key role in the osteogenesis (Nakashima et al., 2002; Komori, 2006). RUNX2 guides lineage commitment of MSCs to osteogenesis instead of adipogenesis and chondrogenesis. RUNX2, osterix, and β-catenin regulate osteoblast terminal differentiation (Komori, 2006). Moreover, the MAF bZIP transcription factor (MAF) was upregulated in osteogenic MSCs. MAF promotes osteogenesis synergized with RUNX2 and suppresses adipogenesis by restraining peroxisome proliferator activated receptor gamma (PPARγ) that directs adipogenic lineage commitment of MSCs (Nishikawa et al., 2010). The core-binding factor subunit beta (CBFβ) forms a complex with RUNX2 to activate the Wnt/β-catenin pathway and repress adipogenesis-related gene expression, as a result, maintaining osteogenesis and suppressing adipogenesis (Nishikawa et al., 2010). Moreover, forkhead box P1 (FOXP1) interacts with CCAAT enhancer binding protein beta (CEBPβ) required for adipogenesis, and represses the activation of the NOTCH pathway to promote biased osteogenesis and inhibit adipogenesis (Li et al., 2017b).

### 5.2 Effect of Growth Factors/Peptides on Osteogenesis and Chondrogenesis of Stem Cells Laden on the 3D-Printed Hydrogel

As aforementioned, some growth factors/peptides were mixed into the hydrogel to induce the osteogenesis and chondrogenesis of 3D scaffold-loaded stem cells. As the classical growth factors are widely used in bone tissue engineering, BMPs attract great focus and have been proven by the Food and Drug Administration (FDA). Poldervaart et al. (2013). designed a BMP-2 sustained release system loaded in the stem cell-laden hydrogel-based 3D construct, and the continuous release of BMP was detected in a high level during 3 weeks culture while the increased osteogenesis and bone formation were observed in the mice and rat models. In addition, assembling BMP-2 into the scaffold is another strategy to maintain the sustained release. Du et al. (2015) assembled a kind of collagen-binding domain-bone morphogenetic protein 2 (CBD BMP-2)-collagen microfibers, and this hydrogel mixed with the BM-MSCs were printed as the bioink. Similarly, stable release of BMP was observed under a confocal microscope, and osteogenic differentiation of BM-MSCs was more effective. BMP-7, a member of the BMP family that could regulate the early staged signals of osteogenesis and bone formation, was immobilized on the 3D-printed hydrogel, and greatly promoted osteogenic differentiation *via* the activation of SMAD (Lee et al., 2018). To address the issue of fast degradation of the BMPs, Park et al. (2020) developed a synthetic BMP-2 mimetic peptide and tethered it into hydrogel before bioprinting, which showed robust acceleration of the hDPSC osteogenic differentiation. For cartilage regeneration, TGF-β1 was tethered to a HA-based bioink to induce the chondrogenic differentiation of BM-MSCs, showing increased chondrogenic gene expression, ECM deposition, and activated early TGF-β1 signaling (Hauptstein et al., 2022). In a rabbit osteochondral cartilage defect model, an interleukin-4 (IL-4)-loaded hydrogel-based 3D-printed scaffold was implanted, with 8- and 16-week observation, and the IL-4-loaded scaffold significantly promoted the formation of neocartilage and neobone tissues according to the assessment of safranin-O, Col II immunohistochemical staining, and micro-CT (Gong et al., 2020).

## 6 Conclusion and Perspective

3D bioprinting refers to a promising and an innovative technique that can be used to fabricate the 3D scaffold with the bioink composed of cells and materials and demonstrates great potential in creating complex tissue constructs (Jeon et al., 2022). Hydrogels are deemed ideal and appealing materials with regard to its controllable biological and biophysical properties to construct a desirable 3D-bioprinted ECM for the attachment, proliferation, migration, and differentiation of the cells loaded in (Gao et al., 2019). Diverse kinds of cells have been implied in bone and cartilage tissue engineering while chondrocytes and stem cells are the most potential cell types among them. Although chondrocytes are the intrinsic cells of the cartilage and could repair the osteochondral damages, the limited supply, long expansion time, and the possibility of differentiation into fibroblast restrict the application of them. On the other hand, the abundant source, the easy isolation procedure, the large quantity of the stem cells, and the suitable biological characteristics including the adjustable differentiation into osteoblasts and chondrocytes greatly broaden the clinical prospect (Xu et al., 2020). Meanwhile, the advances of the stem cell therapy in the regenerative medicine stimulated the progress of the stem cell-laden hydrogel as the bioink of 3D bioprinting. As a result, the 3D bioprinting technique and hydrogels combine with stem cells that could better meet the demand of the clinical requirement and have significantly facilitated the regeneration of bone and cartilage tissue engineering over the past decade (Ni et al., 2020; Buyuksungur et al., 2021; Ge et al., 2021; Huang et al., 2021; Nedunchezian et al., 2021; Jeon et al., 2022).

Stem cell-laden hydrogel-based 3D bioprinting has provided new opportunities for bone and cartilage tissue repair. Backbone of this system consists of biomaterials, stem cells, bioprinting methods, and their interactive microenvironment. Compared with conventional 2D tissue engineering, 3D bioprinting optimizes the structural and living conditions of stem cells. Over the past decades, emerging research studies have focused on the advancement of this technique regarding to its core elements to make it a substitute or better alternative over autologous bone and cartilage tissue. However, current strategies are still of limitations.

**FIGURE 4:**
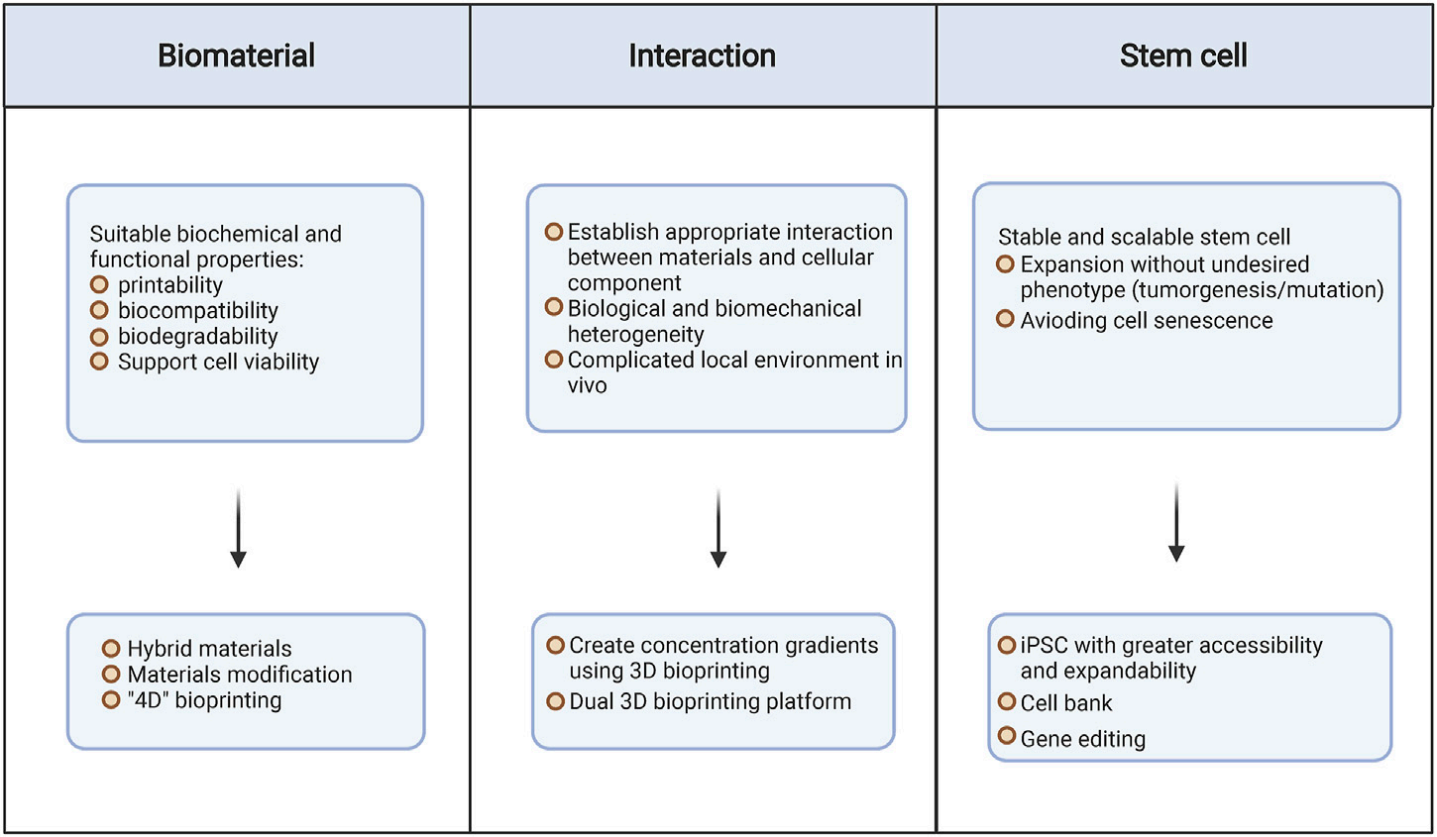
Current challenges and solutions for bone and cartilage tissue engineering.

Constructing biomaterial with suitable biochemical and functional properties (such as printability, biocompatibility, biodegradability, and maintenance of cell viability) remains challenging (Figure 4). Some features of natural biomaterials may hinder their ideal usage in 3D bioprinting. For example, the natural alginate shows superior biocompatibility and biodegradability but lacks sufficient mechanical strength (Jang et al., 2018). Hybrid materials may help to overcome such limitation by producing more versatile items. For example, alginate hydrogel could maintain long-term strength after cross-linking with Ca^2+^, while gelatin could stabilize the 3D structure at the primary stage; hence, alginate–gelatin-mixed hydrogel has been extensively researched in tissue engineering because of the ideal biocompatibility and the improved mechanical property (Axpe and Oyen, 2016; Chawla et al., 2020). Meanwhile, adding Col I to agarose significantly increases cell spreading and differentiation (Duarte Campos et al., 2016). However, this is not the ultimate solution. Though combination of two kinds of hydrogel could make up the shortcoming, they are still far away from the requirement of the product. For instance, low-density alginate/gelatin (0.8% alginate)-mixed hydrogel-based scaffold demonstrates great priority in facilitating mineralization, maintaining cell viability, and inducing osteogenic differentiation of BM-MSCs, but the lack of mechanical property, shape fidelity, and printability is inevitable. On the contrary, the high-density alginate/gelatin (1.8% alginate) based-3D printing exhibits improved mechanical features and printability, but largely impairs the cell viability (Zhang et al., 2020c).

A further challenge is to establish appropriate interaction between materials and the cellular component. 3D bioprinting provides space for cell encapsulation and deposition, but lacks the control of biological and biomechanical heterogeneity. Thus, the microenvironment may be inappropriate for cell growth and differentiation into desired cartilage or bone tissue. Solutions for this limitation are to create concentration gradients using 3D bioprinting, for example, the mixing nozzles that can print materials with tunable gradients (Ober et al., 2015). In some cases, the printing patterns are more complicated, such as vascularized bone compositing with the hard mineral structure and the soft organic matrix. Cui et al. have developed a dual-3D bioprinting platform consisting with a fused deposition modeling 3D bioprinter and a stereolithography 3D bioprinter. With the addition of regional immobilization of bioactive factors, the printed system support controlled and provided continuous stimulus for vascularized bone regeneration (Cui et al., 2016). Notably, such interaction may vary in different individuals, influenced by the local destructional area, inflammatory factors, oxygen, *etc*. Therefore, in addition to the improvement of biomaterials, we should also focus on the interaction between biomaterials and cellular components, especially the *in vivo* model.

Selection and production of appropriate stem cell is another challenge for stem cell-laden hydrogel-based 3D bioprinting. Bone and cartilage are mesodermal-derived tissue and thus the multipotent stem cell populations are important source for their biofabrication. Stem cell has multiple differentiation potential and immune advantages over adult cells, but it demands regenerative ability to the ideal phenotype. It has been noticed that MSCs tend to display a hypertrophic phenotype that leads to endochondral bone formation (Eslaminejad and Malakooty Poor, 2014). Thus, the control of regenerative tendency is demanding. Otherwise, different types of stem cell exhibit dissimilar propensity of differentiation in certain scaffold. For instance, BM-MSCs tend to differentiate into chondrogenic cells and osteogenic cells in gelatin while AD-MSCs prone to osteogenesis. Notably, stem cell expansion *in vitro* is complicated. Cell senescence is inevitable following multiple expansion rounds (De Coppi et al., 2007). As a cell candidate, iPSC has shown greater accessibility and expandability. However, the risk of tumorigenicity and mutations has limited its clinical application (Lee et al., 2013; Simonson et al., 2015). The attempt to establish an iPSC bank may help to overcome this issue. Meanwhile, novel protocols for primary cell isolation and expansion are highly requested to support cell sourcing.

In addition, evolution in printing technique advances the bioprinting. Three-dimensional bioprinting produces scaffold that provides spatial environment closer to human tissue for stem cells. However, the current 3D bioprinting cannot meet the diverse needs simultaneously because the scaffold is fixable upon designing and printing. In 2014, Skylar Tibbits demonstrated the 4D printing which counted time as a new dimension (Wan et al., 2020). By adopting stimuli-responsive materials, 4D printing scaffold is flexible and able to change their configuration in response to different stimuli. Its usage in cell-laden bioprinting substantially improves the adjustment and function of implant. We propose that 4D bioprinting is an upcoming trend for bone and cartilage tissue engineering. Despite the high cost of stimuli-responsive and programmable biomaterials, stem cell-laden hydrogel-based 4D bioprinting are opening new avenue for bone and cartilage repair.

## Glossary

3D: Three-dimensional
ABS: Alcian blue staining
AD-MSCs: Adipose-derived mesenchymal stem cells
AGG: Aggrecan
ALP: Alkaline phosphatase
AMPs: Amorphous magnesium phosphates
ARS: Alizarin red staining
BaG: Bioactive glass
BCP: Biphasic calcium phosphate
BMP-2: Bone morphogenetic protein-2
BMP-7D: Bone morphogenetic protein -7-derived peptides
BM-MSCs: Bone marrow-derived mesenchymal stem cells
β-TCP: Beta-tricalcium phosphate powder
BTE: Bone tissue engineering
CB[6]: Cucurbit[6] uril
CD: Chondrogenic differentiation
CNF: Cellulose nanofibrils
Col I: Collagen type I
COL 1A1: Collagen type I alpha 1
Col II: Collagen type II
Col X: Collagen type X,
CTE: Cartilage tissue engineering
DAH: Diaminohexane
DHT: Dehydrothermal
DMD: Digital micro-mirror device
DMMB: Dimethylmethylene blue
DP-MSCs: Dental pulp mesenchymal stem cells
DPSCs: Dental pulp stem cells
eBM-MSCs: Equine bone marrow-derived mesenchymal stem cells
ECM: Extracellular matrix
ESCs: Embryonic stem cells
FGF: Fibroblast growth factor
GAG: Glycosaminoglycans
GelMA: Methacrylated gelatin
GNPs: Gold nanoparticles
HA: Hyaluronic acid
HAMA: Hyaluronic acid methacrylate
H&E staining: Hematoxylin and eosin staining
HAP: Hydroxyapatite
hAD-MSCs: Human adipose-derived mesenchymal stem cells
hBM-MSCs: Human bone marrow-derived mesenchymal stem cells
hBM-MStCs: Human bone marrow-derived mesenchymal stromal cells
hMSCs: Human mesenchymal stromal cells
HPMC-MA: Hydroxy propyl methyl cellulose of methacrylation
hUCB-MSCs: Human umbilical cord blood-derived mesenchymal stem cells
hTMSCs: Human turbinate-derived mesenchymal stromal cells
ICC: Immunocytochemistry
IHC: Immunohistochemistry
Ma-dECM: Methacrylated decellularized extracellular matrix
MAF: MAF bZIP transcription factor
MeHA: Methacrylated hyaluronic acid
MPEG-b-PCL: Methoxy PEG-block-PCL
MSCs: Mesenchymal stem cells
MTS: Masson’s trichrome staining
NA: Not available
OCN: Osteocalcin
OD: Osteogenic differentiation
OMA: Oxidized and methacrylated alginate
OPF: Oligo(poly(ethylene glycol) fumarate
OPN: Osteopontin
OTE: Osteochondral tissue engineering
P(AGE-co-G): Poly(allyl glycidyl ether-co-glycidyl)
PBMCs: Peripheral blood mononuclear cells
PCL: Polycaprolactone
PEG: Poly(ethylene glycol)
PLA: Polylactic acid
PLGA: Poly(lactic-co-glycolic acid)
PSRS: Picrosirius red staining
PVA: Polyvinyl alcohol
rBM-MSCs: Rat bone marrow-derived mesenchymal stem cells
raBM-MSCs: Rabbit bone marrow-derived mesenchymal stem cells
RUNX 2: Runt-related transcription factor 2
sGAG: Sulfated glycosaminoglycans
SOX9: SRY box transcription factor 9
TGF-β: Transforming growth factor-beta
UVECs: Umbilical vein endothelial cells
UV: Ultraviolet
VEGF: Vascular endothelial growth factor
